# Monitoring cellular redox dynamics using newly developed BRET-based redox sensor proteins

**DOI:** 10.1016/j.jbc.2021.101186

**Published:** 2021-09-10

**Authors:** Nae Fu, Kazunori Sugiura, Kumiko Kondo, Shungo Nakamura, Ken-ichi Wakabayashi, Toru Hisabori

**Affiliations:** 1 School of Life Science and Technology, Tokyo Institute of Technology, Yokohama, Japan; 2 Laboratory for Chemistry and Life Science, Tokyo Institute of Technology, Yokohama, Japan

**Keywords:** biosensor, BRET, cyanobacteria, photosynthesis, ROS, redox regulation, AMS, 4-acetamido-4′-maleimidylstilbene-2,2′-disulfonate, BRET, bioluminescence resonance energy transfer, cDNA, complementary DNA, CFP, cyan fluorescent protein, DCMU, 3-(3,4-dichlorophenyl)-1,1-dimethylurea, EGFP, enhanced GFP, EM-CCD, electron-multiplying charge-coupled device, eNL, enhanced Nano-lantern, ER, endoplasmic reticulum, MV, methyl viologen, Ni–NTA, nickel– nitrilotriacetic acid, ROS, reactive oxygen species, YFP, yellow fluorescent protein

## Abstract

Reactive oxygen species are key factors that strongly affect the cellular redox state and regulate various physiological and cellular phenomena. To monitor changes in the redox state, we previously developed fluorescent redox sensors named Re-Q, the emissions of which are quenched under reduced conditions. However, such fluorescent probes are unsuitable for use in the cells of photosynthetic organisms because they require photoexcitation that may change intracellular conditions and induce autofluorescence, primarily in chlorophylls. In addition, the presence of various chromophore pigments may interfere with fluorescence-based measurements because of their strong absorbance. To overcome these problems, we adopted the bioluminescence resonance energy transfer (BRET) mechanism for the sensor and developed two BRET-based redox sensors by fusing cyan fluorescent protein–based or yellow fluorescent protein–based Re-Q with the luminescent protein Nluc. We named the resulting *r*ed*o*x-sensitive *B*RET-based *in*dicator probes “ROBINc” and “ROBINy.” ROBINc is pH insensitive, which is especially vital for observation in photosynthetic organisms. By using these sensors, we successfully observed dynamic redox changes caused by an anticancer agent in HeLa cells and light/dark-dependent redox changes in the cells of photosynthetic cyanobacterium *Synechocystis* sp. PCC 6803. Since the newly developed sensors do not require excitation light, they should be especially useful for visualizing intracellular phenomena caused by redox changes in cells containing colored pigments.

Reactive oxygen species (ROS) are secondary products from aerobic metabolisms such as the mitochondrial and photosynthetic electron transport chains (1, 2). Because ROS oxidizes biological components such as proteins, nucleic acids, and lipids, cells possess several ROS scavenging systems (3). Conversely, at low concentrations, ROS are critical signaling factors (3, 4). Therefore, cells have various mechanisms to control the ROS concentration and the redox state of each intracellular compartment (5, 6). Especially in photosynthetic organisms, light conditions govern the intracellular redox states and regulate various enzyme activities (7). Therefore, time-lapse observations of redox state changes in living cells are crucial for understanding the redox regulation systems in the cell and the physiological phenomena caused by redox state changes.

Therefore, to date, several redox-sensitive fluorescent proteins have been developed. rxYFP (8) was developed by introducing cysteine pair at appropriate positions, N149 and S202, on the surface of the yellow fluorescent protein (YFP) (9). In an oxidizing environment, this cysteine pair forms a disulfide bond that facilitates chromophore protonation and reduces its fluorescence intensity. The roGFP1 and roGFP2 (10) are modified forms of *Aequorea victoria* GFP (11, 12) and enhanced GFP (EGFP) (13, 14), respectively. They carry a cysteine pair at positions S147 and Q204. These roGFP proteins have two excitation peaks derived from protonated and deprotonated chromophores at 400 nm and 475 to 490 nm, respectively. Similar to rxYFP, the oxidation of cysteine pairs promotes the protonation of their chromophores. Therefore, the fluorescence intensity ratio of two excitation peaks changes according to the dissociation and formation of the disulfide bond. The reversibility of the disulfide bond formation on the rxYFP and roGFP molecules enables real-time imaging of the redox state in living cells. The midpoint redox potentials of rxYFP, roGFP1, and roGFP2 are −261, −288, and −272 mV, respectively, at pH 7 (8, 10). These values are suitable for redox imaging in the cytosol of mammalian cells (−320 mV at pH 7.0) (15). These sensors monitor the intracellular redox state primarily determined by the balance between the reduced and oxidized glutathione (8).

The conformational change in the β-barrel structures of fluorescent proteins because of the introduced Cys pair also affects fluorescence quantum yields, as reported for the reversible redox-sensitive fluorescent probes Oba-Q (16) and Re-Q (17). The fluorescence intensities of Oba-Q and Re-Q decrease under oxidized and reduced conditions, respectively. Re-Qc is derived from the cyan fluorescent protein (CFP) mTurquoise (18), and Re-Qy1 is derived from the YFP Venus (19). These probes also have two cysteine residues at positions S147 and Q204 and several mutations near C147. The emission peaks and midpoint redox potentials of Re-Qc and Re-Qy are 480 nm and −286 mV, and 525 nm and −263 mV, respectively (17). Because these probes do not require the chromophore hydroxyl group, the CFP-based probe, in particular, has better pH stability than GFP-based or YFP-based probes. A disadvantage of these probes is that signal intensities are directly affected by protein expression levels because the ratiometric analyses do not apply to this type of sensor.

Although fluorescent protein–based sensors are helpful for nondestructive and continuous observations in living cells, excitation lights for these probes sometimes excite endogenous pigments in the cell that may cause phototoxicities (20, 21). In particular, the excitation light effects would be critical when the fluorescent probes are applied to the photosynthetic cells. In addition, various chromophore molecules in the photosynthetic cells have strong absorption properties, which interfere with fluorescence-based measurements. Some sensors employ the bioluminescence resonance energy transfer (BRET) mechanism to overcome this problem (22, 23, 24). In 2016, Suzuki *et al.* (25) developed luminescent proteins, named “enhanced Nano-lantern (eNL),” by fusing Nanoluc (Nluc, the brightest luciferase mutant derived from the *Oplophorus* luciferase), with various colored fluorescent proteins. Some eNL-based probes were used to monitor intracellular functions such as Ca^2+^ dynamics (25). We also successfully monitored the *in vivo* pH dynamics in cyanobacteria *Synechocystis* sp. PCC 6803 (*Synechocystis*) using a BRET-based probe, Luphin (26).

In this study, we developed BRET-based redox probes by fusing CFP-based and YFP-based Re-Q sensors with Nluc to observe redox state changes. We named these protein probes ROBINc and ROBINy, respectively. ROBIN stands for “*R*ed*o*x-sensitive *B*RET-based *in*dicator.” Using these sensors, we successfully detected redox state changes in both mammalian cells and cyanobacteria.

## Results

### Development of BRET-based redox probes

**Figure 1:**
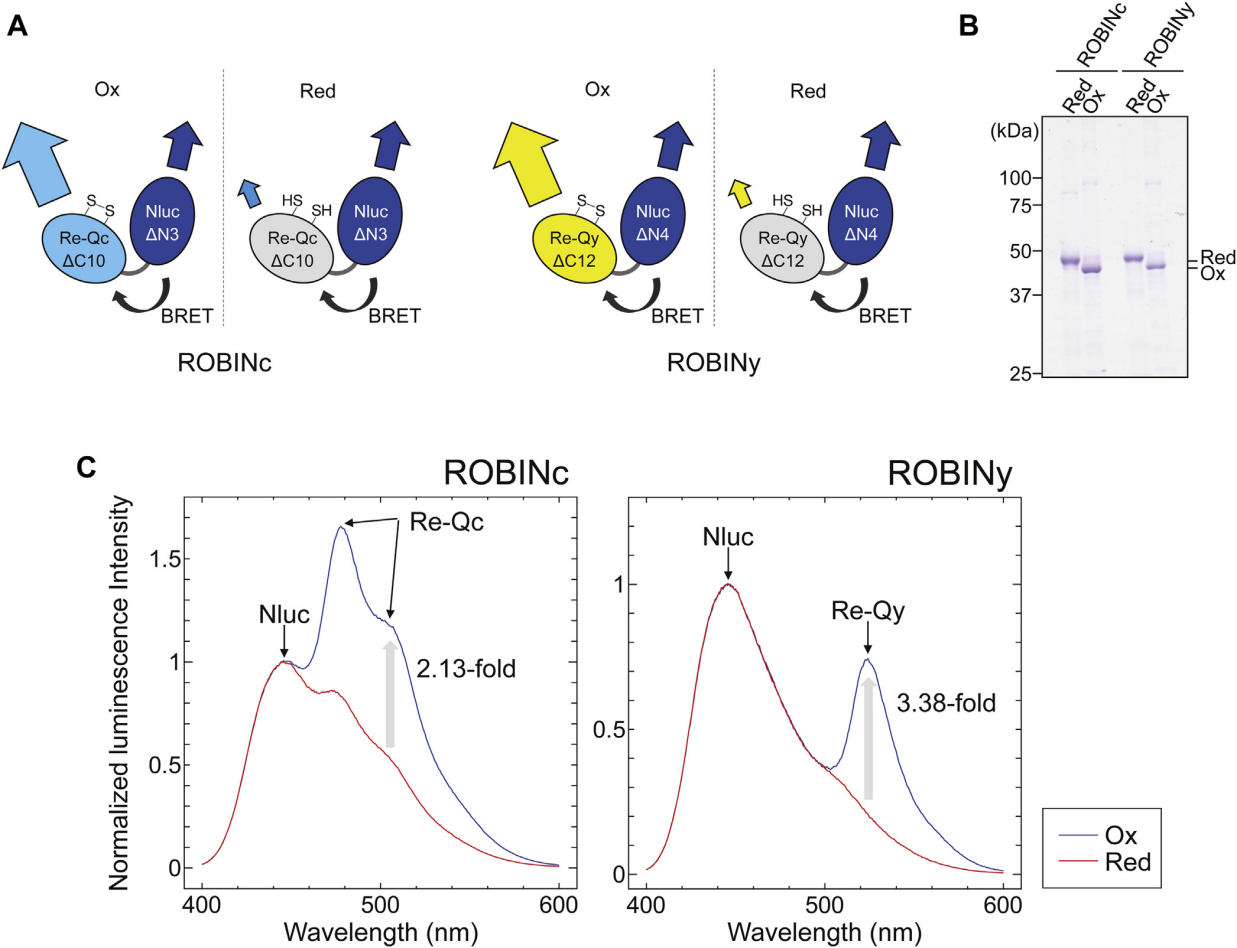
**Molecular designs of ROBINc and ROBINy.***A*, schematics of ROBINc and ROBINy. Re-Qc or Re-Qy is fused to Nluc with a linker sequence comprising two amino acids. *B*, confirmation of oxidation (ox) and reduction (red) of ROBINc and ROBINy by AMS labeling and nonreducing SDS-PAGE. *C*, the luminescence spectra of ROBINc (*left*) and ROBINy (*right*) at 25 °C in 50 mM Tris–HCl (pH 7.0). Probes were oxidized using 0.5 mM diamide and reduced using 5 mM DTT. The luminescence spectra were normalized to the maximum luminescence value of the first peak of Nluc at 445 nm. The emission peaks at 477 to 478 nm and 524 to 525 nm were derived from Re-Qc and Re-Qy, respectively. AMS, 4-acetamido-4′-maleimidylstilbene-2,2′-disulfonate.

To obtain bioluminescent redox-sensitive protein probes, we fused Nluc and Re-Q. In the presence of its substrate furimazine, Nluc, an efficient BRET donor (25, 27), emits a strong luminescence signal with a peak at 450 nm. We used two redox-sensitive fluorescent probes Re-Qc and Re-Qy as BRET acceptors (17). In addition, we introduced two mutations (G145S and W146F) to the original Re-Qc to improve its dynamic range. Herein, “Re-Qc” refers to this new mutant and “Re-Qy” refers to Re-Qy1 from our previous article (17). In the case of the CFP-Nluc type probe ROBINc, we connected Re-QcΔC10 (Re-Qc lacking C-terminal 10 amino acids) with NlucΔN3 *via* the linker amino acid sequence Pro-Tyr. In contrast, the YFP type probe ROBINy comprises Re-QyΔC12, the linker amino acid sequence Glu-Leu, and NlucΔN4 (Fig. 1*A*). The redox states of cysteine pairs on these probes were confirmed by the mobility shift on SDS-PAGE with the labeling of the free thiol group with 4-acetamido-4′-maleimidylstilbene-2,2′-disulfonate (AMS) (Fig. 1*B*).

Both ROBIN probes had two emission peaks derived from Nluc and Re-Q proteins. Under oxidizing conditions, the relative signal intensities from Re-Q were dramatically higher than those under reducing conditions (Fig. 1*C*, *blue lines*). Switching from reducing conditions to oxidizing conditions increased the Re-Q/Nluc ratios of ROBINc and ROBINy by 2.13-fold and 3.38-fold, respectively. When excitation and emission spectra of these probes were measured (ROBINc: Ex [excitation maximum wavelength] = 430 nm and Em [emission maximum wavelength] = 480 nm, ROBINy: Ex = 510 nm and Em = 525 nm), they showed strong fluorescence in the oxidized form (Fig. S1*A*), which is a typical spectral property of Re-Q. In contrast, changing the redox conditions did not affect the absorption spectra of ROBINc (Fig. S1*B*, *left panel*) but affected that of ROBINy (Fig. S1*B*, *right panel*).

### Characterization of ROBINc and ROBINy *in vitro*

**Figure 2:**
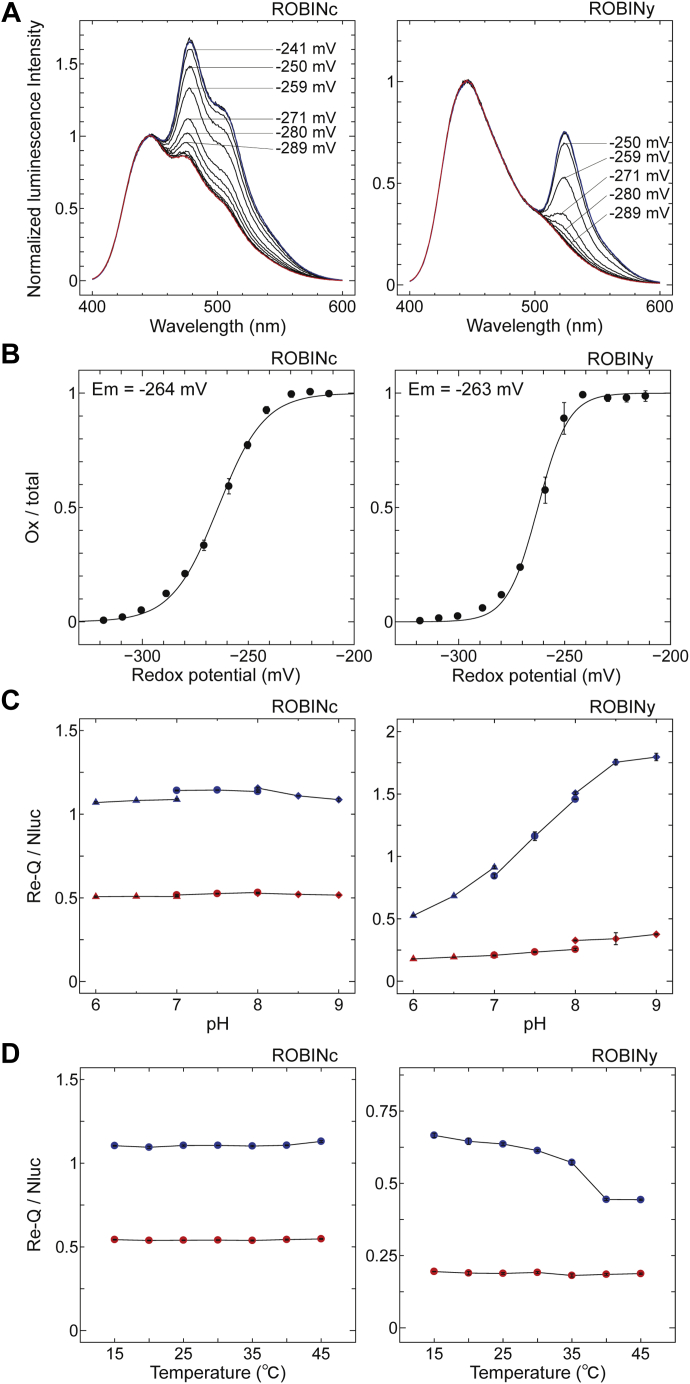
**Characterization of ROBINc (*left*) and ROBINy (*right*).***A*, luminescence spectra of ROBIN probes in DTT buffers with various redox potentials. *B*, redox titration of ROBINc and ROBINy. The oxidation levels of ROBINc and ROBINy were quantified by calculating the ratio of oxidized form to total proteins and plotted against the calculated redox potentials of DTT buffers (pH 7.0). Data were fitted to the Nernst equation. *C*, effect of the pH on the Re-Q/Nluc ratios of ROBINc and ROBINy. ROBINc and ROBINy were oxidized with 0.5 mM diamide or reduced with 5 mM DTT, and sum of the signal intensity from 495 to 540 nm for the emission signal of Re-Qc, that from 515 to 560 nm for the emission signal of Re-Qy, and that from 420 to 460 nm for the luminescence signal of Nluc, was measured at 0.5-nm intervals, respectively; 50 mM buffers Pipes (for pH 6–7, *closed triangle*), Hepes (for pH 7–8, *closed circle*), and tricine (for pH 8–9, *closed diamond*) were used. Then, Re-Q/Nluc ratios of oxidized (*blue symbols*) and reduced (*red symbols*) forms were calculated. *D*, effect of the temperature on Re-Q/Nluc ratios of ROBINc and ROBINy. Re-Q/Nluc ratios of oxidized (*blue symbols*) and reduced (*red symbols*) forms were measured according to the conditions shown in *C*. Measurements were performed after incubation of probes at each temperature for at least 40 min.

Midpoint redox potentials of these probes were determined using the luminescence spectra measured under DTT solutions with different redox potentials (Fig. 2*A*). The obtained midpoint redox potentials of ROBINc and ROBINy at pH 7.0, determined from plots of fractions of oxidized probes against redox potentials, were −264 and −263 mV, respectively (Fig. 2*B*).

Next, the stability of the signals of ROBIN probes against pH and temperature changes was examined. As shown in Figure 2*C* (*left panel*) and Figure 2*D* (*left panel*), Re-Q/Nluc ratios of ROBINc were stable within the physiologically relevant pH range (6, 7, 8, 9) and from 15 to 45 °C. However, ROBINy was pH stable in the reduced form but not in the oxidized form (Fig. 2*C*, *right panel*). The observed pH dependency might be owing to the pH sensitivity of the YFP chromophore. Although the luminescence from Re-Qy decreased under high-temperature conditions, its dynamic range was larger than that of ROBINc (Fig. 2*D*, *right panel*). Therefore, especially between pH 8 and 9, ROBINy must be useful for measuring the change in redox state under stable pH conditions.

**Figure 3:**
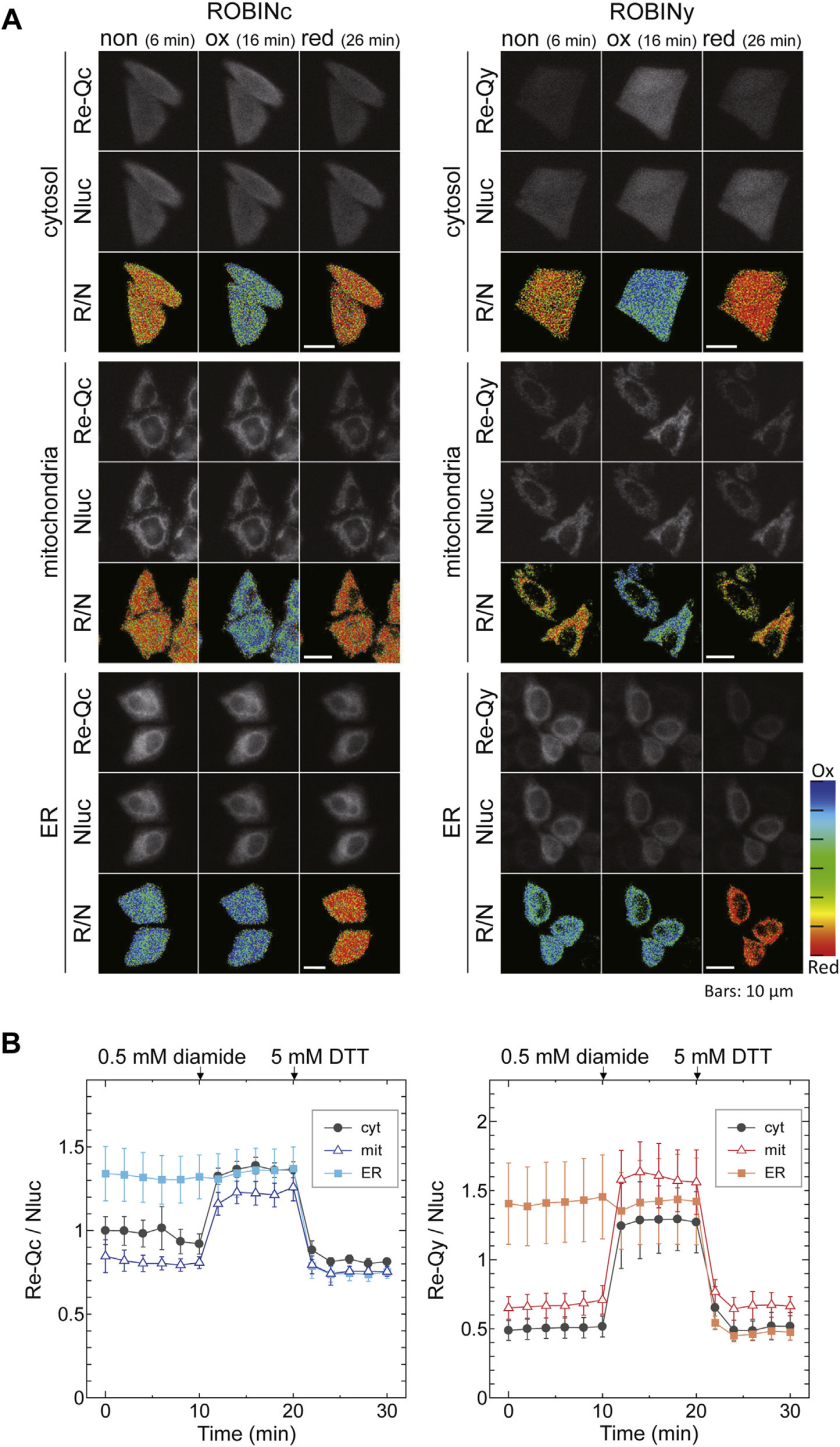
**Luminescence redox responses of ROBINc and ROBINy in HeLa cells.***A*, luminescence images of the HeLa cells expressing ROBIN probes at the Re-Q range (Re-Qc: 495–540 nm, Re-Qy: 515–560 nm), at the Nluc range (420–460 nm), and Re-Q/Nluc ratios calculated at the indicated periods. For the changes in redox state of the cell, 0.5 mM diamide was added at 10 min and 5 mM DTT was added at 20 min. The scale bar represents 10 μm. *B*, Re-Q/Nluc ratios plotted against time. Signal intensities of the images of 29 cells for cyt-ROBINc, 34 cells for mit-ROBINc, 22 cells for ER-ROBINc, 69 cells for cyt-ROBINy, 31 cells for mit-ROBINy, and 30 cells for ER-ROBINy were calculated and averaged.

We then performed the measurements *in vivo*. HeLa cells expressing ROBINc or ROBINy in the cytosol, mitochondria, and endoplasmic reticulum (ER) were prepared. Using a microscope with an electron-multiplying charge-coupled device (EM-CCD) camera, we measured the signal intensities of the probes in the cells under standard culture conditions; Re-Q/Nluc ratios of ROBINc were 0.91 ± 0.06 in the cytosol and 0.81 ± 0.03 in the mitochondria. These values suggest that in both locations, the reduced form of ROBINc was predominant. However, adding 0.5 mM diamide (an oxidant) rapidly increased the Re-Q/Nluc ratio (cytosol: 1.36 ± 0.04, mitochondria: 1.26 ± 0.03). Adding 5 mM DTT (a reductant) decreased the Re-Q/Nluc ratios to 0.81 ± 0.02 in the cytosol and 0.75 ± 0.03 in the mitochondria (Fig. 3*A*, left, and Fig. 3*B*, *left*; *circle* and *triangle*). By contrast, ROBINc in ER showed high Re-Q/Nluc ratios under both standard culture conditions (1.32 ± 0.13) and in the presence of 0.5 mM diamide (1.37 ± 0.13), indicating that ROBINc was fully oxidized in the ER. The addition of DTT certainly reduced ROBINc in the ER (Fig. 3*A*, *left* and Fig. 3*B*, *left*; *square*). Although ROBINy showed similar changes in the luminescence intensity ratio as ROBINc (Fig. 3*A*, *right* and Fig. 3*B*, *right*), the observed redox indicator signal Re-Q/Nluc ratio did not show the same trend when the changes for each organelle were compared. This may be due to the fact that the Re-Qy signal is more sensitive to changes in pH. The redox state in mitochondria should also have a strong effect on their pH. These results suggest that these probes, especially ROBINc, can detect organelle-specific differences in the redox state of HeLa cells.

**Figure 4:**
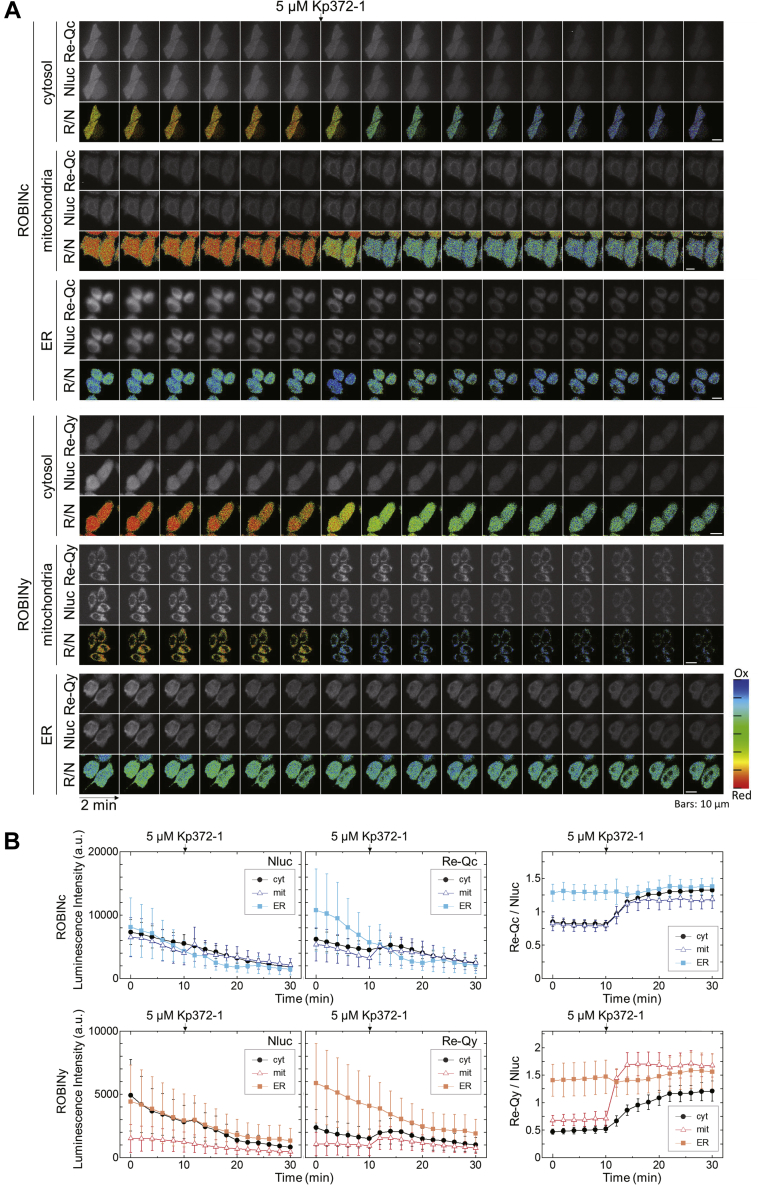
**ROS induction imaging in HeLa cells.***A*, luminescence images of HeLa cells expressing ROBINc (*top*) and ROBINy (*bottom*) at the Re-Q range (Re-Qc: 495–540 nm, Re-Qy: 515–560 nm), at the Nluc range (420–460 nm), and Re-Q/Nluc ratios calculated at the indicated times with 2-min intervals. Kp372-1 was added to make the final concentration of 5 μM at 10 min. The scale bars represent 10 μm. *B*, luminescence intensity at the Nluc region (*left panel*), that at the Re-Q region (*center panel*), and the Re-Q/Nluc ratios (*right panel*) are indicated. To obtain these values, luminescence intensities of 52 cells for cyt-ROBINc, 33 cells for mit-ROBINc, 19 cells for ER-ROBINc, 35 cells for cyt-ROBINy, 41 cells for mit-ROBINy, and 24 cells for ER-ROBINy were calculated and averaged. ROS, reactive oxygen species.

Next, we examined whether these probes could detect cellular redox changes caused by ROS generation. We treated the HeLa cells expressing ROBIN probes with Kp372-1 (5 μM) (Fig. 4, *A* and *B*). Kp372-1 is an anticancer drug candidate, which is reduced by NAD(P)H dehydrogenase in tumor cells and activates ROS release (28). As shown in Figure 4, ROBINc and ROBINy expressed in the cytosol and mitochondria of HeLa cells were slowly oxidized in the presence of 5 μM Kp372-1, whereas no redox state change occurred in the ER.

### Redox changes in cyanobacteria *Synechocystis* sp. PCC 6803

Because the photosynthetic reaction changes intracellular pH, we expressed pH stable ROBINc in *Synechocystis* cells in order to observe the intracellular redox changes (26). We then confirmed that the cells expressed the desired probe using Western blotting analysis (Fig. S2). The expression of ROBINc(SS), a redox-insensitive mutant protein whose 147th and 204th cysteines are replaced with serines, was also confirmed.

To confirm the usefulness of the luminescent type sensor in photosynthetic cells, we first checked whether the fluorescent signal of the Re-Q domain of ROBINc could be applied to the measurement of redox changes in cells. The *Synechocystis* cells contain various chromophores in the cell. Especially, carotenoids have strong absorption at the region where Re-Q shows the typical fluorescence changes (Fig. S3, *A* and *B*). In this region, the typical fluorescence spectra of Re-Q sensors are hardly observed (Fig. S3, *C*–*E*). We, therefore, subtracted the fluorescence spectrum of the wildtype cells from that of the ROBINc-expressing cells (Fig. S3*F*) and could obtain a spectrum with the same shape of the recombinant ROBINc. The cells were treated with DTT or diamide for the redox change measurement, and fluorescence spectral changes were obtained (Fig. S3*G*). However, we could not determine whether the fluorescence changes in this region were linear because the spectra of the wildtype cells and those of ROBINc(SS) mutant expressing cells also showed unexpected changes caused by treatments with redox reagents (Fig. S3*G*). These results imply that the Re-Q part alone is not suitable for observing redox changes in photosynthetic cells.

**Figure 5:**
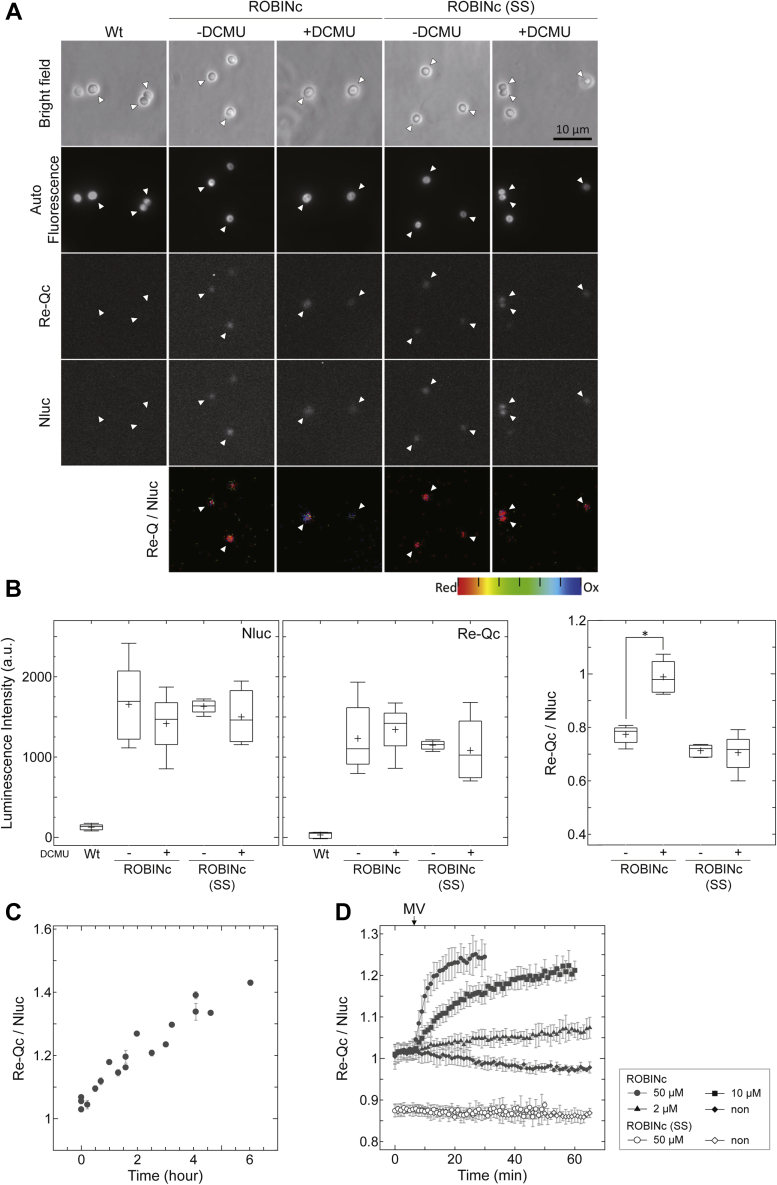
**Redox response imaging in *Synechocystis*.***A*, luminescence images of *Synechocystis* expressing ROBINc, ROBINc(SS), and the wildtype cells (Wt), which were obtained at 495 to 540 nm for Re-Qc and 420 to 460 nm for Nluc. Chlorophyll autofluorescence image was obtained above 575 nm with excitation from 530 to 550 nm. The signals from Nluc and Re-Qc were detected in this order with 5-min exposure. The scale bar represents 10 μm. Before the observation, the cells were cultured in the presence or the absence of 10 μM DCMU, a photosynthesis inhibitor, under white light at 40 μmol photons m^−2^ s^−1^ for at least 7 h. The location of the cells is indicated by an *open triangle* in each panel. *B*, luminescence intensity at the Nluc region (*left panel*), that at the Re-Q region (*center panel*), and Re-Q/Nluc ratios (*right panel*). *Center lines* show the medians, and *crosses* represent the means. *C*, dark period–dependent changes in Re-Q/Nluc ratio. The cells in the liquid medium were irradiated with white light at 40 μmol photons m^−2^ s^−1^ for more than 7 h and then placed in the dark. The cells were collected at regular time intervals, and the Re-Q/Nluc ratio was monitored using the plate reader. In each plot, three independent measurement data are averaged and shown with error bars. *D*, Re-Q/Nluc ratios measured after MV addition at 1-min intervals in *white light* (40 μmol photons m^−2^ s^−1^). The redox states were monitored using the plate reader. And results obtained from three (ROBINc(SS), *open symbols*) or four (ROBINc, *closed symbols*) independent experiments are shown with error bars. DCMU, 3-(3,4-dichlorophenyl)-1,1-dimethylurea; MV, methyl viologen.

We, therefore, observed the luminescence signals from *Synechocystis* cells expressing ROBINc under a microscope with an EM-CCD camera in the absence and the presence of 10 μM 3-(3,4-dichlorophenyl)-1,1-dimethylurea (DCMU), a photosynthesis inhibitor, and detected signals derived from Nluc and Re-Qc with an exposure time of 5 min (Fig. 5*A*). We calculated the Re-Qc/Nluc ratios as described in the Experimental procedures section (Fig. 5*B*). When the cells were cultured under white light (40 μmol photons m^−2^ s^−1^) in advance, the Re-Q/Nluc ratio of ROBINc was 0.77 ± 0.03 (Fig. 5*B*). By contrast, the ratio increased to 0.99 ± 0.06 when *Synechocystis* cells were cultured in the presence of DCMU (Fig. 5*B*). However, in *Synechocystis* cells expressing ROBINc(SS), DCMU did not affect the Re-Q/Nluc ratio (without DCMU: 0.71 ± 0.02, with DCMU: 0.71 ± 0.06).

Next, we measured the luminescence change in the cell cultures using a plate reader (Tristar LB941; Berthold). The cells were transferred from light to dark, and the luminescence change was measured (Fig. 5*C*). The results showed that the Re-Qc/Nluc ratio changed almost in proportion to the length of the dark period. However, when using the cell mixture directly, the self-absorption of intracellular pigments disturbed the measurement, as expected. Therefore, we compared the luminescence change signal (Re-Qc/Nluc ratio) with the oxidation level of ROBINc at the indicated time points, which we determined using Western blotting after AMS labeling (Fig. S4*A*). Consequently, the oxidation level of ROBINc was linearly proportional to the Re-Q/Nluc ratio, and we could use the obtained regression line to estimate the intracellular oxidation level (Fig. S4*B*). Note that the chlorophyll molecules absorb most luminescence signal of Nluc, causing a significant difference between the Re-Q/Nluc ratio measured with the plate reader (corresponding to the oxidation level of ROBINc) and the values obtained from recombinant proteins or single-cell observations. Consequently, the Re-Q/Nluc ratio is higher than those values.

Finally, we observed the intracellular changes in redox state under photosynthetic conditions by adding methyl viologen (MV) to *Synechocystis* cells (Fig. 5*D* and Fig. S5, *A* and *B*). MV is a ROS inducer in photosynthetic organisms (29). Irradiating the cells with white light (40 μmol photons m^−2^ s^−1^) in the presence of MV increased the Re-Q/Nluc ratio of ROBINc, implying a change in cellular ROS levels (Fig. 5*D*, *closed symbols*). The oxidation level plotted against the elapsed time after the addition of MV was fitted by the regression line, and the maximum oxidation level and the time constant were then obtained based on Equation 1.

(1) $$y=\left(Max\times x\right)/\left(t_{1\text{/}2}+x\right)+\;{\text{a}}_{\text{0}}$$

where *y* is the oxidation level, *Max* is the maximum oxidation level, *x* is the time after the addition of MV, and a_0_ is the value of the oxidation level of ROBINc before the addition of MV. Within 20 min of the addition of 50 μM MV, the oxidation level of ROBINc reached approximately 50% (Fig. S5*C*). In this case, the time constant *t*_1/2_ was 5.32 ± 1.99 min, whereas it was 23.38 ± 8.49 with 10 μM MV (Table 1). By contrast, the Re-Q/Nluc ratios were always stable regardless of the addition of MV in *Synechocystis* cells expressing ROBINc(SS) (Fig. 5*D*, *open symbols*). Hence, the new sensor allows kinetic analysis of the intracellular redox changes.

**Table 1:** Redox changes in cyanobacterial cells caused by MV

MV (μM)	Max (ox/total)	*t*_1/2_ (min)
50	0.50 ± 0.04	5.32 ± 1.99
10	0.48 ± 0.03	23.4 ± 8.49
2	0.17 ± 0.03	61.9 ± 31.7

The maximum oxidation level (Max) and the half time (*t*_1/2_) were determined from the results shown in Figure 5*D*.

## Discussion

In this study, we developed two luminescent redox-sensitive probes, ROBINc and ROBINy, by using the redox sensor Re-Q (17) and the luminescent protein Nluc based on the molecular design of eNL (25), in which CeNL lacks C-terminal 10 amino acids of CFP and YeNL lacks C-terminal 12 amino acids of YFP. In order to obtain the high FRET efficiency, not only the overlap of donor and acceptor spectra but also the exciton orientation is very important. However, it is known that the C-terminal side of fluorescent protein is very flexible and disordered in the crystal structure. Therefore, it was necessary to remove the C-terminal side of CFP and YFP proteins to limit the rotation angle within the fusion protein molecule. Moreover, we varied linker sequences and selected a linker that anchored the two components in a good orientation and showed high FRET efficiency. The linker sequence between Re-Q and Nluc was determined based on the results of direct selection of the *Escherichia coli* colony spots expressing the randomly mutated sensor proteins, which showed the highest Re-Q/Nluc signal ratio. Both have two emission peaks derived from Re-Q and Nluc, which allow us to quickly estimate the intracellular redox states using the emission intensity ratio Re-Q/Nluc (Fig. 1*C*). The results shown in Fig. S1, *A* and *B* suggest that the emission intensity change of ROBINc is due to the emission intensity changes of the Re-Qc domain, which is dependent on the redox state. In ROBINy, both the emission intensity changes, originating from the change in the absorption spectrum of Re-Qy and the change in the BRET efficiency, affected the change in the luminescence spectrum.

The BRET efficiency depends on the overlap integral of the donor emission spectrum with the acceptor absorption spectrum and the distance and orientation between these domains (30, 31). Because redox-sensitive cysteines are present only in the Re-Q domain, we expected the distance and orientation between the two domains to remain unchanged. Furthermore, the shape of the absorption spectrum did not change, and we suggest that the emission intensity changes of ROBINc were caused by emission intensity changes of the Re-Qc domain, which depend on redox states. In ROBINy, both the emission intensity changes of Re-Qy and BRET efficiency changes caused by absorption spectrum changes affected luminescence (Fig. S1, *A* and *B*).

Glutaredoxin is a small protein containing CPYC motif as an active center, and glutathione is the most abundant thiol compound in cells and plays an important role in the maintenance of intracellular redox balance (32, 33, 34, 35). *Synechocystis* sp. PCC 6803 has three glutaredoxin genes (two class I genes and one class II gene) (36). Among them, grxB, one class I glutaredoxin gene, is expressed constantly, whereas grxA, a class I gene, and grxC, a class II gene, are particularly expressed under stress conditions in this cyanobacterium (37). In this study, we designed Re-Q (the redox-sensitive sensor domains of ROBINc and ROBINy) based on the molecular design of roGFP and rxYFP (17). Because the referenced sensors roGFP and rxYFP were reported to monitor the redox states of the glutathione–glutaredoxin system *in vivo* (8, 16), the observed oxidations of ROBIN in cyanobacterial cells should also reflect changes in the intracellular glutathione redox states through endogenous glutaredoxin, especially class I glutaredoxin.

ROBINc and ROBINy had midpoint redox potentials of −264 and −263 mV, respectively (Fig. 2, *A* and *B*). These are less negative than that of roGFPs (−288 to −272 mV) (10) and almost the same as that of rxYFP (−261 mV) (8). The results imply that midpoint redox potentials of these sensors are also less negative than the reported redox potentials in the nucleus (−340 mV) (38), cytosol (−320 mV) (15), and mitochondria (−360 mV) (10) of HeLa cells. The application of the sensors must be therefore limited under certain suitable conditions. When we applied the FROG/B sensor to the cytosol of the filamentous cyanobacteria *Anabaena* sp. PCC7120, the redox potentials were −298 to −291 mV under light conditions and were more negative than −265 mV under dark conditions at pH 7.0 (35). As shown in Figure 5*C*, when the redox state change of ROBINc expressed in *Synechocystis* cells was induced by the light/dark transition, the midpoint redox potential of about −287 to −281 mV under light conditions and more negative than −260 mV under dark conditions was estimated. We, therefore, concluded that ROBINc could be reliably applied to the measurement of the intracellular redox state of cyanobacterial cells, although there might be some differences among different species of cyanobacteria. In addition, when ROBINc and ROBINy were localized to cytosols and mitochondria of HeLa cells, these sensors could catch redox state changes in these compartments (Fig. 4). However, the higher midpoint redox potential in ER (−210 mV) fully oxidizes the probes, rendering them inoperative in this compartment under standard culture conditions (Fig. 4) (39).

Recently, luminescence imaging tools were used for follow-up observations of tumor tissues *in vivo* (40, 41). These luminescent redox probes might allow us to gather information on oxidized stress levels in tumor tissues and confirm the effects of anticancer agents *in vivo*, as shown in Figure 4.

Besides, BRET-based probes do not require excitation lights because they use luciferin–luciferase reactions for excitation. This crucial characteristic makes them applicable to photosynthetic cells. Indeed, we successfully measured the intracellular pH changes in cyanobacterial cells under photosynthetic conditions owing to the BRET-based pH sensor (26). Moreover, based on the previous findings (26), we noticed that the pH insensitivity of ROBINc is also important for observations in cyanobacteria. The broad temperature stability of ROBINc is also suitable for various culture conditions of photosynthetic organisms. For instance, the usual laboratory species of *Chlamydomonas* grow well at 20 to 25 °C (42), and the optimal growth temperature range for *Synechocystis* is 32 to 38 °C (43). Furthermore, high- and low-temperature stress in higher plants generates ROS (44, 45). In wheat, for example, high-temperature stress conditions induce the expression of 2-Cys Prx, a ROS scavenger (46). Therefore, ROBINc might be useful for monitoring the redox status in various photosynthetic organisms.

Although we successfully monitored the light/dark-dependent redox state changes in *Synechocystis* cells using ROBINc, this probe has some demerits. First, the photosynthetic inhibitor DCMU strongly affected the cellular redox state (Fig. 5, *A* and *B*). DCMU inhibits NADP reductions, which may explain this change (47). However, these single-cell measurements using ROBINc required prolonged exposure (5 min/frame) because an EM-CCD camera cannot detect weak emission intensities from each cell. Therefore, tracing fast redox state changes occurring on a scale of seconds to minutes using this sensor protein is challenging. The ROBIN sensor protein consumes the specific substrate furimazine for the luminescence reaction, gradually decreasing the luminescence signals in a time-dependent manner. Next, we attempted to detect the luminescent signals from ROBIN sensors using a plate reader to reduce measurement time. Consequently, it allowed us to detect strong signals from multiple cells with an exposure time of 0.5 s/frame and measure the cytosolic oxidations caused by 50 μM MV with sufficient temporal resolution (Fig. 5*D*). MV decreased the luminescence intensities of probes in a concentration-dependent manner (Fig. S5*A*). This phenomenon must be because MV causes an electron transfer from the photosystem to molecular oxygen, which decreases the oxygen amount (29). However, the ratio values of Re-Q/Nluc are independent of those emission intensities in principle. In addition, Re-Q/Nluc values of the negative control ROBINc(SS) were stable (Fig. 5*D*, *open symbols* and Fig. S5*B*). Therefore, we concluded that the change in the emission intensity ratio because of MV is solely due to the redox change.

As mentioned previously, MV exhibits rapid ROS generation because it effectively receives electrons from photosystem I and reduces oxygen (29). By monitoring the Re-Q/Nluc ratios, we successfully observed the MV-dependent dynamics of ROS evolution (Fig. 5*D*). The time constant of ROS generation was determined using the time variation of the Re-Q/Nluc ratio (Fig. S5*C* and Table 1), indicating the usefulness of the sensor proteins to monitor the *in vivo* redox dynamics.

Redox dynamics play pivotal roles in various metabolic processes occurring in phototrophs, and GSH:GSSG redox state contributes to the overall intracellular redox environment, rather than determining the environment. In the photosynthetic cells, the GSH:GSSG ratio must be directly affected by the photosynthetic conditions. It will also be strongly impacted by ROS. In addition, the very low concentrations of ROS are also involved in the stomatal movement (48), pollen tube growth (49, 50), and root hair growth (51, 52). As shown in this report, ROBIN sensors could detect the intracellular redox changes assisted by glutaredoxin promptly. Because the BRET-type redox probe does not require the excitation light source, it will contribute to the visualization of intracellular phenomena caused by redox changes in cells containing colored pigments such as photosynthetic organisms.

## Experimental procedures

### Gene construction

To prepare the expression plasmid for ROBINc, complementary DNAs (cDNAs) of Re-QcΔC10 and NlucΔN3 were amplified by PCR and fused using the hot-fusion method (53). Saturated mutations were introduced in two amino acids of the linker region in the vicinity of redox responsive cysteine pairs. Finally, we selected the linker sequence Pro-Tyr and the G145S/W146F mutations for their high BRET efficiency and large dynamic ranges against redox state changes. For ROBINy, we amplified the cDNAs of Re-QyΔC12 and NlucΔN4 using PCR and fused them with the random linker sequence comprising two amino acids. The Glu-Leu linker was finally selected for ROBINy. The cDNA fragments encoding the mature protein-coding regions were cloned into pET-23a vector (Novagen) to express the protein with a C-terminal His tag.

### Protein expression and purification

*E. coli* strain BL21(DE3) was transformed with each expression plasmid. Transformed *E. coli* was cultured at 37 °C to obtain an absorbance value of 0.3 to 0.6 at 600 nm. Next, the protein expressions were induced by the addition of 0.5 mM IPTG (final concentration), and the cells were incubated at 21 °C overnight. The expressed proteins were purified by nickel– nitrilotriacetic acid (Ni–NTA) affinity chromatography using Ni–NTA agarose (Qiagen) resin. Ni–NTA agarose resin was washed using 50 mM Tris–HCl (pH 8.0) containing 20 mM imidazole, and the proteins were eluted with the same buffer containing 250 mM imidazole. The elutions were then applied to a TOYOPEAL Butyl-650 column (TOSOH) and eluted using 50 mM Tris–HCl (pH 7.0) with an ammonium sulfate reverse gradient from 20% to 0%. Peak fractions containing recombinant proteins were collected, dialyzed for removing ammonium sulfate using 50 mM Tris–HCl (pH 7.0) buffer, and concentrated.

### Fluorescence spectroscopy

Fluorescence excitation/emission spectra were determined using a fluorescence spectrophotometer, FP-8500 (JASCO). For measurements, excitation wavelengths of 430 and 510 nm and emission wavelengths of 480 and 525 nm were used for ROBINc and ROBINy, respectively. Absorbance spectra were measured using a spectrophotometer, V-650 (JASCO).

For oxidation and reduction, probes were incubated for 40 min in the presence of 0.5 mM diamide and 5 mM DTT_red_, respectively. For excitation/emission spectral measurement, 1 μM recombinant proteins in 50 mM Tris–HCl (pH 7.0) buffer were used, and for absorbance spectral measurements, 10 μM proteins were used.

### Luminescence spectroscopy

Luminescence spectra were measured using an FP-8500 fluorescence spectrophotometer. The excitation light was terminated, and the emission intensity at each wavelength was measured. Integrated luminescence intensities of sensors from 420 to 460 nm (Nluc), from 495 to 540 nm (Re-Qc), and from 515 to 560 nm (Re-Qy) were calculated from the emission spectra.

Fully oxidized and reduced probes were prepared similar to that in fluorescence spectroscopy. Sample solutions containing 5 nM probes, 0.2% (v/v) furimazine of Nano-Glo luciferase assay system (Promega), and 50 mM Tris–HCl (pH 7.0) were used.

### Determination of the midpoint redox potential

ROBIN probes (5 μM) were incubated in 50 mM Tris–HCl (pH 7.0), 20 mM DTT_ox_, and various concentrations of DTT_red_ (from 8 μM to 20 mM) overnight at 25 °C. Oxidation ratio, Ox/total, was calculated using Equation 2.

(2) $$\text{Ox}/\text{total}=\left(\text{R}-{\text{R}}_{\text{red}}\right)/\left({\text{R}}_{\text{ox}}-{\text{R}}_{\text{red}}\right)$$

where R is the Re-Q/Nluc ratio for each sample, and R_ox_ and R_red_ are those at fully oxidized conditions and fully reduced conditions, respectively. The midpoint redox potential values of probes were calculated by fitting the titration data of Ox/total to the Nernst equation according to the methods described in our previous study (54). A value of −330 mV was used as the midpoint redox potential of DTT at pH 7.0 (55).

### Imaging of redox states in HeLa cells

ROBIN probes were expressed in HeLa cells using modified pEGFP-N1 (Addgene) as an expression vector. Expression vectors were generated by replacing the gene for EGFP with that for ROBINc or ROBINy. In addition, Lipofectamine 3000 (Life Technologies) was used as a transfection reagent. After replacing medium containing 0.5% (v/v) furimazine, cells were observed using an Olympus IX73 Inverted microscope and imagemX2, an EM-CCD camera (Hamamatsu Photonics). To obtain emission signals from Nluc, Re-Qc, and Re-Qy, BA420-460, BA495-540GFP, and BA515-560YFP filters (Olympus) were used, respectively. The exposure period was 15 s for each wavelength range. The intensity of the luminescent image was digitalized using the image analysis software ImageJ (NIH).

### *Synechocystis* culture

*Synechocystis* and its probes expressing strain were cultured in BG-11 medium (56) buffered with 20 mM Hepes–KOH (pH 7.4). Cultures were grown at 30 °C under continuous light conditions (40 μmol photons m^−2^ s^−1^) and aerated with 1% (v/v) CO_2_. The cells at the exponential phase were used for the measurement.

### Construction of the *Synechocystis* strain expressing ROBINc probes

ROBINc and ROBINc(SS) were expressed under the control of PsbAⅡ promoter in *Synechocystis*. The cDNAs of ROBINc probes and the pTCP2031V vector were amplified and fused using the hot-fusion method. The pTCP2031V was derived from pTKP2031V (57, 58) and contained a chloramphenicol-resistant cassette instead of a kanamycin-resistant cassette. The resultant plasmid was transformed into *Synechocystis* by homologous recombination. Plasmids selected by DNA sequencing were mixed with *Synechocystis* cells. The transformed *Synechocystis* cells were selected in BG-11 medium containing 20 μg/ml chloramphenicol, and complete segregation of the mutant was confirmed by PCR. ROBINc expression in *Synechocystis* was confirmed by Western blotting.

### Imaging of *Synechocystis*

Cells were observed under a microscope (Olympus IX73) with an EM-CCD camera (imagemX2) in the presence of 2% (v/v) furimazine. To obtain the intensities from Nluc and Re-Qc, BA420-460 and BA495-540GFP filters were used, respectively. The exposure period for each wavelength range was 5 min. The intensity of the luminescent image was then digitalized using the image analysis software ImageJ. For the observation of autofluorescence of chlorophylls, fluorescence mirror unit U-FGW (Olympus) was used.

### BRET measurement of redox states in *Synechocystis*

Luminescence from *Synechocystis* expressing ROBINc probes was measured using Tristar LB941 (Berthold). To obtain the intensities from 420 to 460 nm (Nluc) and from 495 to 540 nm (Re-Qc), luminescence was detected using BA420-460 and BA495-540GFP filters, respectively. The exposure period was 0.5 s for each wavelength range, and the measurement was conducted every 1 min. The sample solution containing 290 μl *Synechocystis* solution and 10 μl furimazine solution (0.5% [v/v] furimazine diluted with BG-11 medium) was used. To prepare the *Synechocystis* solution, the culture medium with absorbance value of 0.1 to 0.2 at 750 nm was adjusted to an absorbance value of 0.1 at 750 nm by BG-11 medium. After 5 min of mixing with furimazine, the measurement was initiated.

### Analysis of the *in vivo* redox states of ROBINc in *Synechocystis*

The redox states in the cells were fixed by adding a final concentration of 10% (v/v) tricarboxylic acid to the *Synechocystis* solutions, and the samples were allowed to stand at 4 °C overnight. Cells were collected by centrifugation and washed with acetone. The precipitate was dissolved in protein extraction/thiol labeling solution (62.5 mM Tris–HCl [pH 6.8], 2% [w/v] SDS, 7.5% [v/v] glycerol, 0.01% [w/v] bromophenol blue, and 2 mM AMS) and labeled with AMS for free thiol groups. *In vivo* redox states of ROBINc were analyzed using Western blotting.

## Data availability

All data are contained within the article and can be shared upon request (thisabor@res.titech.ac.jp).

## Supporting information

This article contains supporting information.

## Conflict of interest

The authors declare that they have no conflicts of interest with the contents of this article.
