# Supplementary material for: Monitoring cellular redox dynamics using newly developed BRET-based redox sensor proteins
Source: J Biol Chem. 2021 Sep 10;297(4):101186. doi: 10.1016/j.jbc.2021.101186 (PMC8487062; doi:10.1016/j.jbc.2021.101186)
Supplement: Supplemental Figures S1–S5 [file mmc1.pdf]

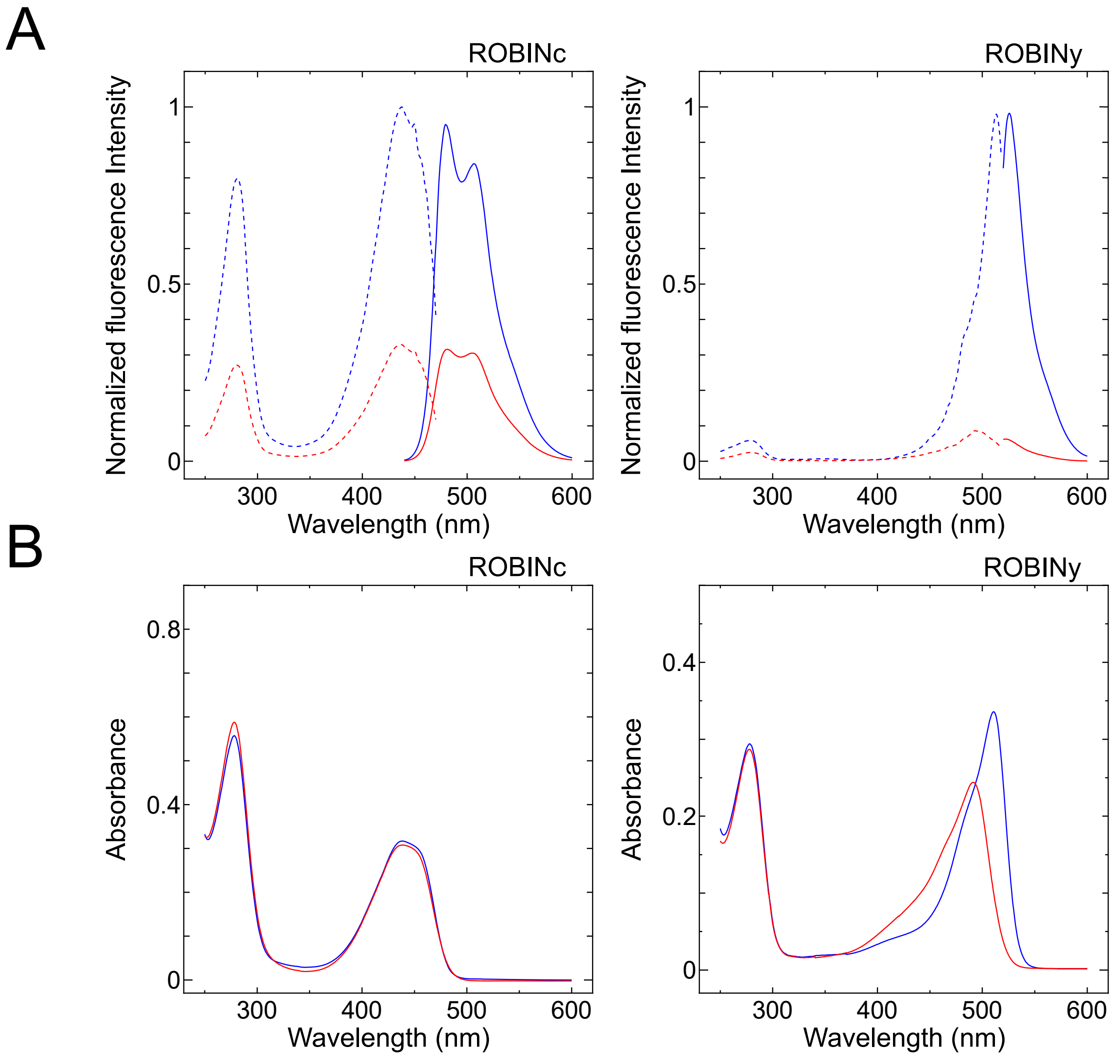

Supplemental Figure 1. Spectrophotometric properties of ROBINc and ROBINy. *A*, excitation (dotted lines) and emission (solid lines) spectra of the oxidized (blue) and reduced (red) forms of ROBINc (left panel) and ROBINy (right panel). *B,* absorption spectra of the oxidized (blue) and reduced (red) forms of ROBINc (left panel) and ROBINy (right panel). Probes were oxidized with 0.5 mM diamide or reduced with 5 mM DTT in advance.

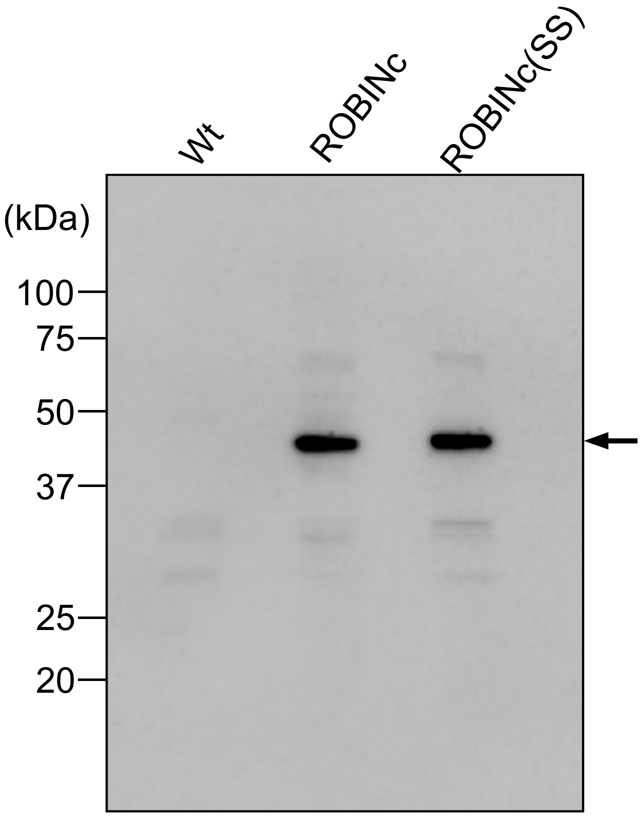

Supplemental Figure 2. Confirmation of ROBINc expression in *Synechocystis*. The expression of ROBINc and ROBINc(SS) in the cytosol of *Synechocystis* was confirmed by western blotting using an antibody against GFP.

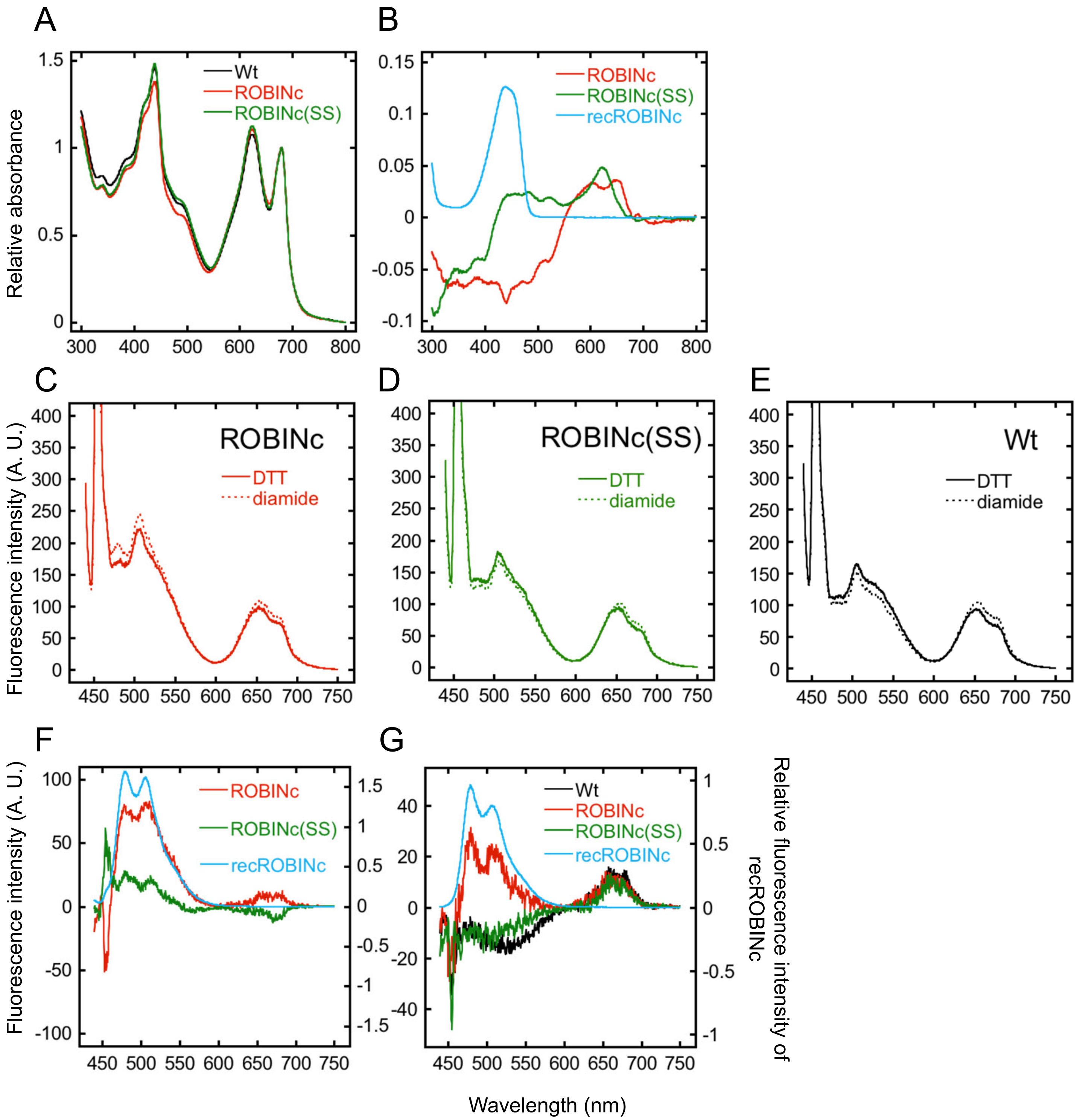

Supplemental Figure 3. Fluorescent signal of the Re-Q part of ROBINc expressed in *Synechocystis*. *A*, the cell cultures of the Synechocystis cells expressing ROBINc and ROBINc(SS), and the wild-type (Wt) cells were prepared with the OD_{750} to be 1.0 and the absorption spectra were measured. Each spectrum was normalized at 680 nm. *B*, the absorption spectrum of the wild-type cells were subtracted from those of ROBIN expressing cells. As a control, the absorption spectrum of the recombinant ROBINc was indicated (light blue line). *C-E*, the fluorescence spectra (Ex = 430 nm) of the Synechocystis cells expressing ROBINc (*C*) and ROBINc(SS) (*D*), and Wt cells (*E*) were measured after reduction (solid line) and oxidation (dotted line). Before the measurements, the cells were prepared with the OD750 to be 2.0, and reduced with 10 mM DTT or oxidized with 1 mM diamide for 1 h on ice. *F*, the fluorescence spectrum of untreated Wt cells were subtracted from those of ROBIN expressing cells. The

fluorescence spectrum of the recombinant ROBIINc (recROBINc) was also indicated as a reference (light blue). *G*, the fluorescent signal of the Re-Q part of ROBINc caused by the redox changes were calculated by the subtraction of the reduced form spectra from the oxidized form spectra; ROBINc (red line), ROBINc(SS) (blue line), and Wt (black line). The difference spectrum of recROBINc was indicated as a reference (light blue line, reduced with 1 mM DTT or oxidized with 0.1 mM diamide for 30 min at 25ºC).

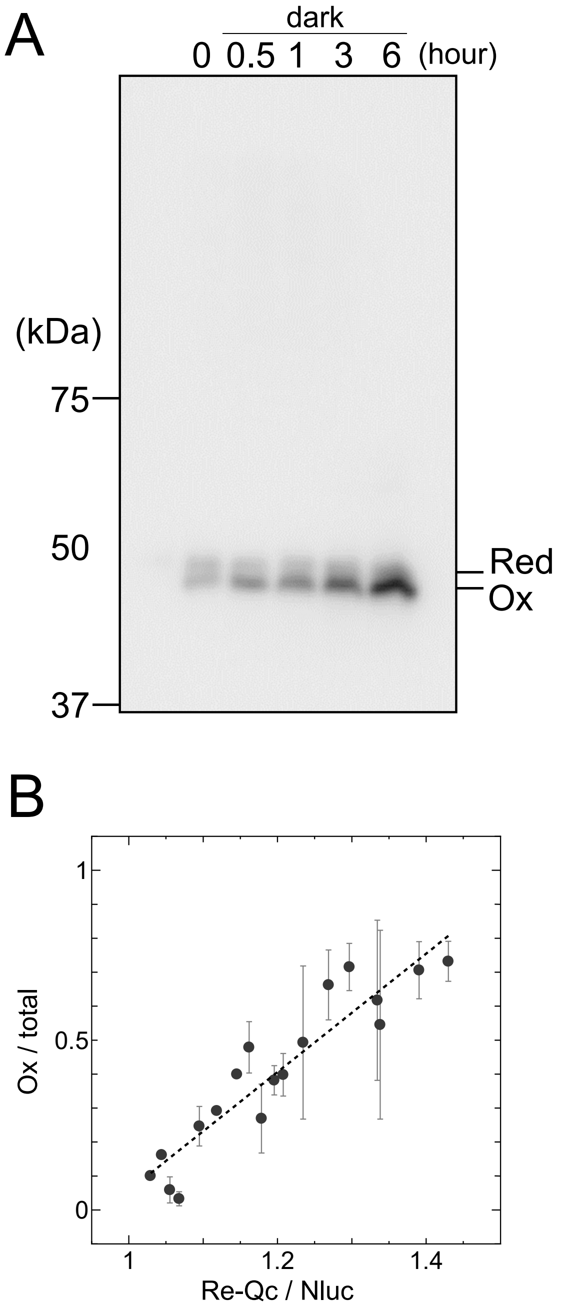

Supplemental Figure 4. The oxidation level analysis of ROBINc in the cells.

A. the *Synechocystis* cells in the liquid medium were treated as shown in the legend to Fig. 5C. Then the cells placed in the dark were collected at the time periods incitated and fixed immediately by TCA, and the oxidation levels of ROBINc was determined by AMS labling and western blotting using an antibody against GFP. *B*, oxidation levels of ROBINc expressed in *Synechocystis* cells against Re-Q/Nluc ratios plot, showing the regression line. Oxidation levels of ROBINc determined in *A* was plotted agains the Re-Q/Nluc ratio of the same sample shown in Fig. 5C, and the regression line was calculated. In each plot, three independent experimental results are averaged and shown with error bars.

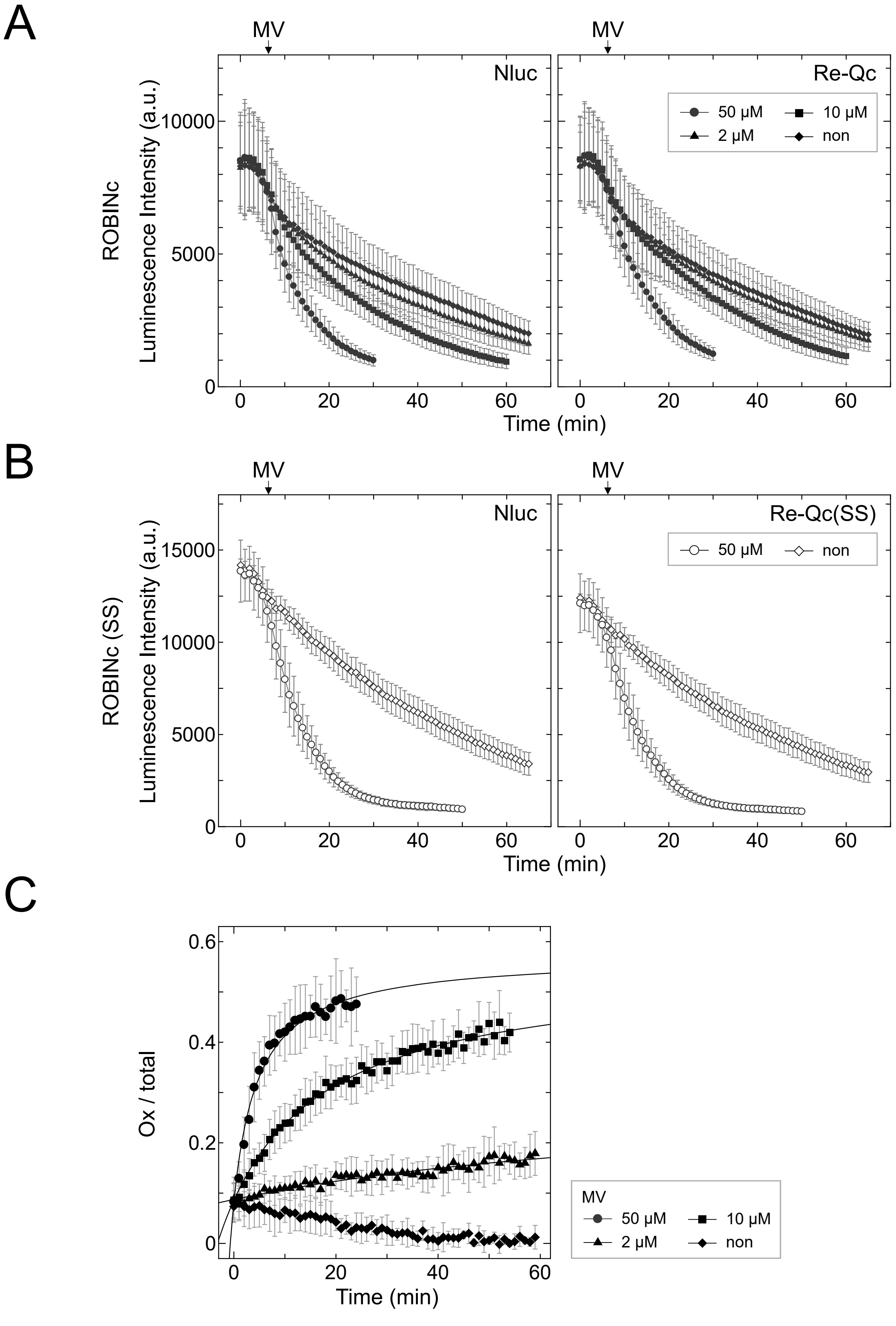

Supplemental Figure 5. Redox changes in cyanobacterial cells caused by MV. *A,* luminescence intensity changes in the Nluc region (left panel) and Re-Q region (right panel) owing to the addition of MV were measured at intervals of 1 min. *Synechocystis* cells expressing ROBINc were irradiated with white light (40 μmol photons m^{-2} s^{-1}). *B, Synechocystis* cells expressing ROBINc(SS) were used to measure the change in luminescence intensity as described in *A*. *C,* oxidation levels (ox/total) were calculated from the data shown in Fig. 5*D*. The regression curves were then calculated for the oxidation levels using Equation [2]. The results obtained in four independent experiments are shown with error bars.
